# Presence of an *Agrobacterium*-Type Tumor-Inducing Plasmid in *Neorhizobium* sp. NCHU2750 and the Link to Phytopathogenicity

**DOI:** 10.1093/gbe/evy249

**Published:** 2018-11-06

**Authors:** Mindia Haryono, Yi-Ming Tsai, Chien-Ting Lin, Fan-Chen Huang, Yan-Chen Ye, Wen-Ling Deng, Hau-Hsuan Hwang, Chih-Horng Kuo

**Affiliations:** 1 Institute of Plant and Microbial Biology, Academia Sinica, Taipei, Taiwan; 2 Department of Life Sciences, National Chung Hsing University, Taichung, Taiwan; 3 Ph.D. Program in Microbial Genomics, National Chung Hsing University and Academia Sinica, Taichung, Taiwan; 4 Department of Plant Pathology, National Chung Hsing University, Taichung, Taiwan; 5 Advanced Plant Biotechnology Center, National Chung Hsing University, Taichung, Taiwan; 6 Innovation and Development Center of Sustainable Agriculture, National Chung Hsing University, Taichung, Taiwan

**Keywords:** *Neorhizobium*, *Agrobacterium*, Ti plasmid, transformation, plant pathogen, comparative genomics

## Abstract

The genus *Agrobacterium* contains a group of plant-pathogenic bacteria that have been developed into an important tool for genetic transformation of eukaryotes. To further improve this biotechnology application, a better understanding of the natural genetic variation is critical. During the process of isolation and characterization of wild-type strains, we found a novel strain (i.e., NCHU2750) that resembles *Agrobacterium* phenotypically but exhibits high sequence divergence in several marker genes. For more comprehensive characterization of this strain, we determined its complete genome sequence for comparative analysis and performed pathogenicity assays on plants. The results demonstrated that this strain is closely related to *Neorhizobium* in chromosomal organization, gene content, and molecular phylogeny. However, unlike the characterized species within *Neorhizobium*, which all form root nodules with legume hosts and are potentially nitrogen-fixing mutualists, NCHU2750 is a gall-forming pathogen capable of infecting plant hosts across multiple families. Intriguingly, this pathogenicity phenotype could be attributed to the presence of an *Agrobacterium*-type tumor-inducing plasmid in the genome of NCHU2750. These findings suggest that these different lineages within the family Rhizobiaceae are capable of transitioning between ecological niches by having novel combinations of replicons. In summary, this work expanded the genomic resources available within Rhizobiaceae and provided a strong foundation for future studies of this novel lineage. With an infectivity profile that is different from several representative *Agrobacterium* strains, this strain may be useful for comparative analysis to better investigate the genetic determinants of host range among these bacteria.

## Introduction

*Agrobacterium tumefaciens* is a soil-dwelling bacterium often associated with plants (Nester 2015). Some strains harbor a tumor-inducing (Ti) plasmid, which is required for their phytopathogenicity. During the infection process, a specific segment of DNA originated from the Ti plasmid (i.e., transfer DNA; abbreviated as T-DNA) is integrated into the plant nuclear genome. The wild-type T-DNA encodes genes for plant hormones auxin and cytokinin, the expression of which would lead to tumor-like cell proliferation, causing crown gall disease. Furthermore, T-DNA also encodes genes for the biosynthesis of opines. The exact type of opine synthesis genes varies and the cognate genes for opine catabolism are located in a separate region of Ti plasmids. Nopaline and octopine are examples of commonly found opines and could be used as a major carbon/nitrogen source for these bacteria (Moore et al. 1997). In other words, through this interkingdom DNA transfer, *A. tumefaciens* could genetically engineer the infected plant and turn its host into a food-producing factory.

One important feature of the *Agrobacterium*-mediated DNA transfer is that none of the genes encoded on the T-DNA is required for transformation. This allows for the replacement of wild-type T-DNA with other genes of interest. Through decades of studies, the major genes involved in T-DNA transfer are now well understood (Gelvin 2003; McCullen and Binns 2006; Kado 2014) and this system has been developed as a critical tool for molecular genetics and biotechnology applications (Bevan 1984; Hood et al. 1986, 1993; Hellens et al. 2000; Lee and Gelvin 2007; Hwang et al. 2017). However, one limitation is that many plant species and cultivars have remained difficult to be transformed by the commonly used strains of *A. tumefaciens*. To overcome this limitation, overexpression of the virulence (*vir*) genes or other modifications have been shown to be effective in some cases (Hellens et al. 2000; Gelvin 2003; Banta and Montenegro 2008; Hiei et al. 2014; Wu et al. 2014; Hwang et al. 2015). As a complementary approach, better sampling of the phenotypic and genomic variations among wild-type strains may further improve our understanding of the genetic mechanisms controlling the host range and transformation efficiency.

In our attempt to isolate and characterize wild-type *Agrobacterium* strains, we obtained a novel strain (i.e., NCHU2750) with a distinct infectivity profile. Moreover, our preliminary genotyping results based on 16S rDNA and *recA* sequences indicated that this strain may not belong to the genus *Agrobacterium* despite their phenotypic resemblance. To better understand this strain, we determined its complete genome sequence and conducted comparative analysis.

## Materials and Methods

### Strain Isolation

The strain NCHU2750 was isolated from a rose gall collected in Changhua County, Taiwan in 2008. The gall was surface-sterilized with 70% ethanol and air-dried in a laminar flow hood for 10 min. After removing the outer layer, tissues were transferred into a grinding bag containing ∼1–3 ml SCPAP buffer (each liter contains 1 g disodium succinate, 1 g trisodium citrate, 1.5 g K_2_HPO_4_, 1 g KH_2_PO_4_, pH 7, the buffer was steam-sterilized, followed by adding 3.52 g filter-sterilized ascorbate and 50 g acid-washed insoluble polyvinylpolypyrrolidone) and ground on ice. The macerated tissue was smeared onto the basal surface of sterilized carrot discs, followed by incubation at 25 °C in a moist chamber until callus formation. The callus was ground in SCPAP buffer and the resulting extract was streaked onto nutrient agar (NA) plates to screen for *Agrobacterium*-like colonies. Colony PCR using *virD1/D2* primers (5′-CGGATCGACGGTTGCTCGCT/5′-CCTGACCCAAACATCTCGGC; PCR product is ∼400 bp) was used for confirmation. Positive samples were purified by streaking single colonies onto new NA plates for three times.

### Genome Sequencing and Analysis

The procedures for genome sequencing and analysis were based on our previous studies (Chung et al. 2013; Lo et al. 2013; Cho et al. 2015; Tsai et al. 2018). All bioinformatics tools were used with the default settings unless stated otherwise. For shotgun sequencing, one paired-end library (∼139-fold coverage) and one mate-pair library (∼298-fold coverage) were prepared and sequenced using the MiSeq platform (Illumina, USA). The de novo assembly was performed using ALLPATHS-LG release 52188 (Gnerre et al. 2011), followed by gap closure and validation using PCR and Sanger sequencing until the complete genome sequence was obtained. The programs RNAmmer (Lagesen et al. 2007), tRNAscan-SE (Lowe and Eddy 1997), and PRODIGAL (Hyatt et al. 2010) were used for gene prediction. The annotation was based on the homologous genes in other genomes (table 1) as identified by OrthoMCL (Li et al. 2003), followed by manual curation based on the KEGG (Kanehisa et al. 2016) and COG databases (Tatusov et al. 2003). The pairwise genome alignments were performed using MUMer v3.23 (Kurtz et al. 2004). The multiple alignment of Ti plasmids was performed using MAUVE v2015-02-25 (Darling et al. 2004).

**Table 1:** Genomic Characteristics of Representative *Neorizobium* and *Agrobacterium* Strains

	*Neorhizobium* sp. NCHU2750	*Neorhizobium galegae* HAMBI 540	*Agrobacterium tumefaciens* C58	*Agrobacterium tumefaciens* Ach5
Accession number	CP030827–CP030833	HG938353–HG938354	AE007869–AE007872	CP011246–CP011249
Genome size (bp)	6,351,242	6,455,027	5,674,258	5,668,655
G+C (%)	60.3	61.2	59.0	58.5
No. of chromosomes	1	1	2	2
Circular	1	1	1	1
Linear	0	0	1	1
No. of chromids	1	1	0	0
No. of plasmids	5	0	2	2
Ti plasmid	+	–	+	+
Protein-coding genes	5,923	6,170	5,355	5,276
rRNA genes	12	9	12	15
tRNA genes	56	51	56	56

For molecular phylogenetics, representative Rhizobiaceae genomes were obtained from GenBank (supplementary table S1, Supplementary Material online). The homologous genes were identified using OrthoMCL (Li et al. 2003). The analyses for 16S rDNA and *recA* were performed using the nucleotide sequences, whereas the analysis for shared single-copy genes was performed using the concatenated protein alignment. The alignment was performed using MUSCLE v3.8 (Edgar 2004), followed by maximum likelihood inference using PhyML v3.0 (Guindon and Gascuel 2003). The proportion of invariable sites and the gamma distribution parameter were estimated from the data set, the number of substitute rate categories was set to four. The bootstrap supports were estimated based on 1,000 replicates.

### Pathogenicity Assays

The procedures for pathogenicity assays were based on our previous studies (Hwang et al. 2010, 2013). The *A. tumefaciens* strains used for comparison include two with a nopaline-type Ti plasmid, C58 (Lin and Kado 1977) and A208 (Sciaky et al. 1978), and three with an octopine-type Ti plasmid, A348 (Garfinkel et al. 1981), Ach5 (Archdeacon et al. 2000), and 1D1609 (Palumbo et al. 1998). Three independent experiments were performed for each assay.

For *Arabidopsis thaliana* ecotype Wassilewskija, the pathogenicity was measured by the transient transformation efficiency on root segments. The binary vector pCAMBIA2201-Gm (Hwang et al. 2010) was introduced into each strain by electroporation, such that the transformation rate could be calculated by counting the percentage of root segments showing GUS activity. For each experiment, at least 10 plants and at least 80 root segments per plant were examined.

For all other plants, the pathogenicity was measured by the tumorigenesis assay. For this assay, 100 μl of 10^9^ CFU/ml bacterial culture was injected into the stem of one-month old plants. After inoculation, the plants were maintained in a greenhouse at 25 °C for one month and then scored for tumors. At least 30 plants were used in each experiment. In addition to the quantitative assays, qualitative confirmation of gall formation on rose was conducted for NCHU2750.

## Results and Discussion

**Fig. 1.**
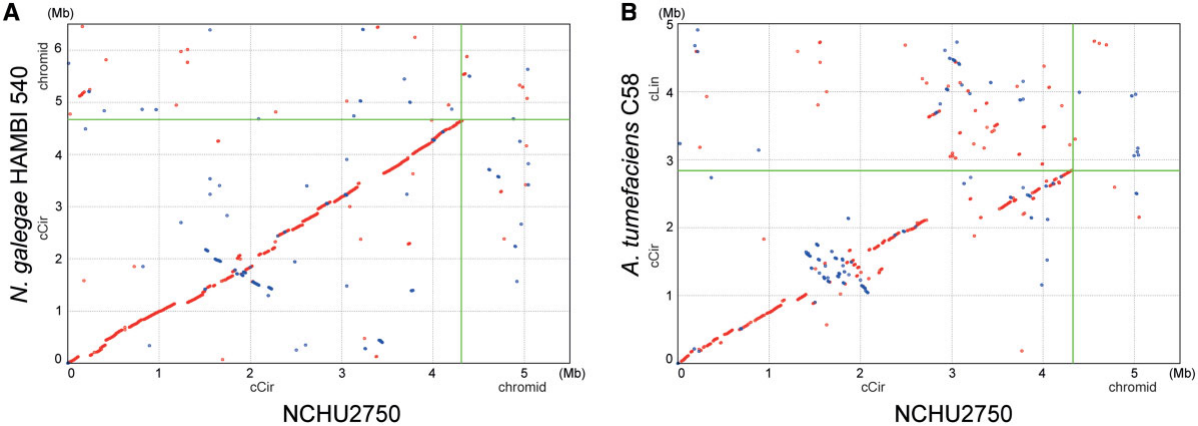
—Pairwise genome alignments between NCHU2750 and (*A*) *Neorizobium galegae* HAMBI 540 and (*B*) *Agrobacterium tumefaciens* C58. Red dots indicate matches on the same strand, blue dots indicate matches on the opposite strands. Abbreviations: cCir, circular chromosome; cLin, linear chromosome.

**Fig. 2.**
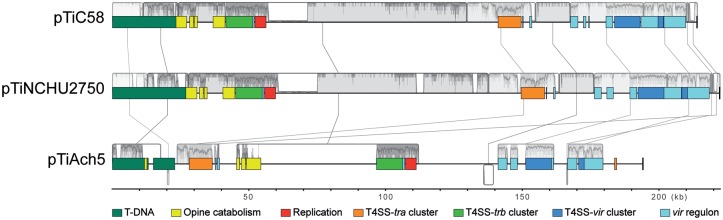
—Multiple alignment of tumor-inducing plasmids (pTi). Regions with high nucleotide sequence identities are indicated by grey boxes and connected by vertical lines. Gene clusters with specific functions are color-coded.

**Fig. 3.**
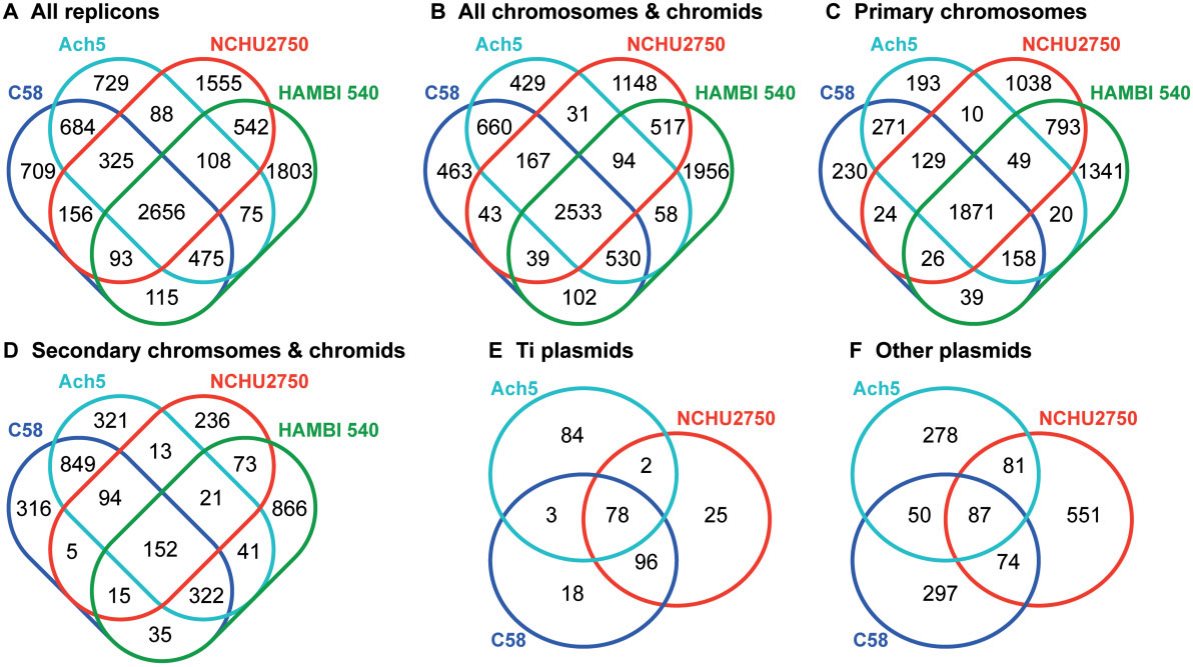
—Venn diagrams showing the numbers of shared and strain-specific homologous gene clusters. (*A*) All replicons. (*B*) All chromosomes and chromids (i.e., excluding plasmids). (*C*) Primary chromosomes (i.e., circular chromosomes of the four strains). (*D*) Secondary chromsomes and chromids (i.e., linear chromosomes of *Agrobacterium* and chromids of *Neorhizobium*). (*E*) Ti plasmids. (*F*) Other plasmids.

The genome of NCHU2750 consists of one 4,319,396-bp circular chromosome, one 764,863-bp chromid (Harrison et al. 2010), one 222,464-bp nopaline-type Ti plasmid, and four other plasmids (table 1). This genome organization is distinct from *A. tumefaciens*, which typically has one ∼2.8–3.1 Mb circular chromosome and one ∼2.1–2.3 Mb linear chromosome (Goodner et al. 2001; Wood et al. 2001; Slater et al. 2009; Wibberg et al. 2011; Slater et al. 2013; Huang 2015; Cho et al. 2018). Rather, NCHU2750 is similar to *Neorhizobium galegae* in its chromosomal organization (Österman et al. 2014). Examination of the synteny further confirmed that the circular chromosome of NCHU2750 exhibits a higher level of conservation with *N. galegae* (fig. 1). Moreover, despite these *Neorhizobium* chromids and *Agrobacterium* linear chromosomes all originated from intragenomic gene transfer from chromosomes to plasmids (Slater et al. 2009), no obvious synteny conservation was observed among these replicons. Alignments among Ti plasmids revealed that pTiNCHU2750 is highly similar to the nopaline-type pTiC58, whereas distinct from the octopine-type pTiAch5 (fig. 2). The patterns based on the distribution of homologous gene clusters are consistent with the levels of synteny conservation among replicons (fig. 3 and supplementary table S2, Supplementary Material online). In short, the strain NCHU2750 has a chromosome that is similar to *N. galegae*, as well as a nopaline-type Ti plasmid that is similar to *A. tumefaciens* C58.

**Fig. 4.**
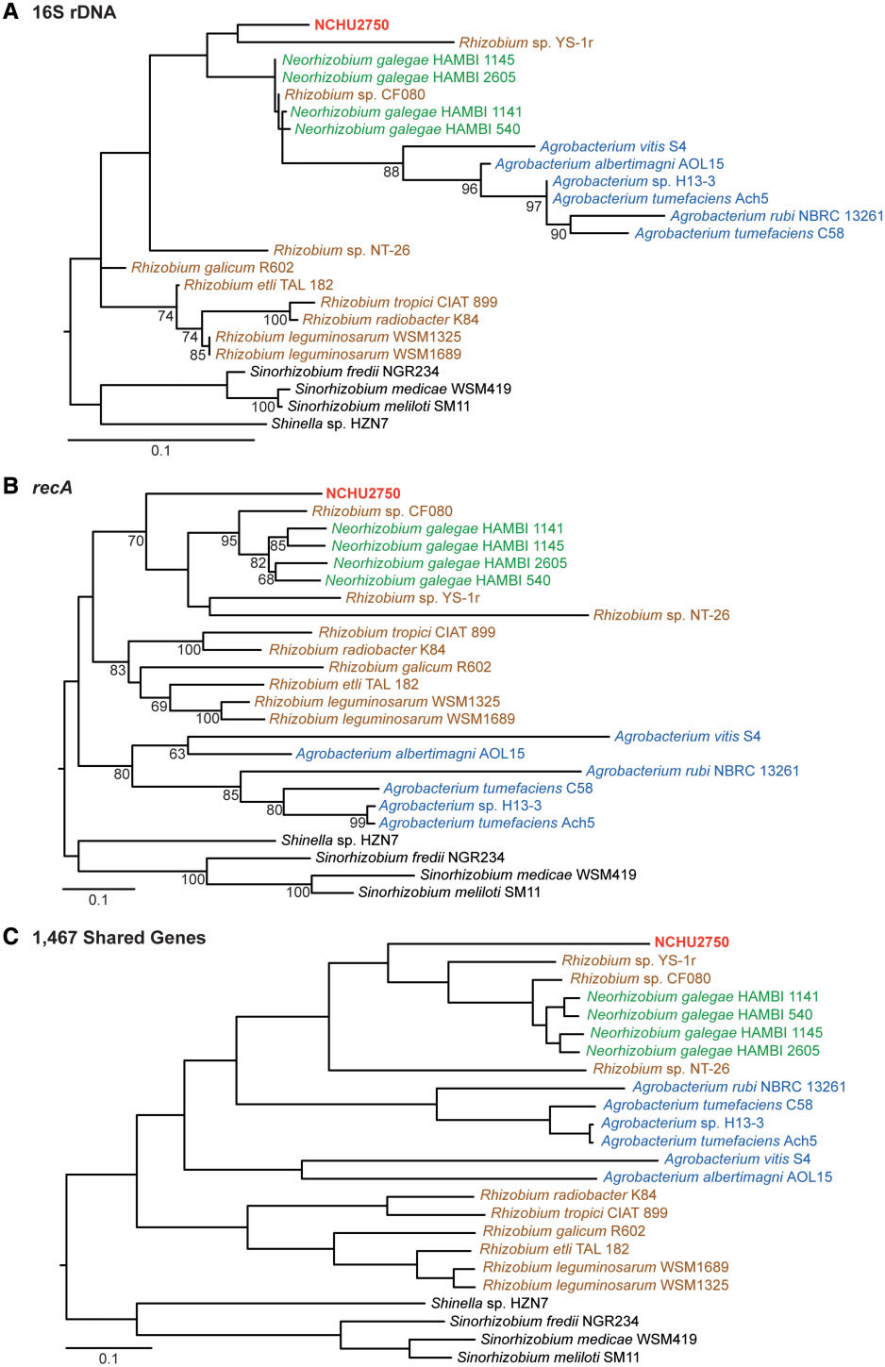
—Maximum likelihood molecular phylogeny. (*A*) and (*B*), nucleotide phylogenies based on 16S rDNA and *recA*, respectively. Bootstrap values above 60 are labeled. (*C*) A protein phylogeny based on a concatenated alignment of 1,467 shared single-copy genes with 512,228 aligned sites. All nodes received 100% bootstrap support.

Results from molecular phylogenetic analysis (fig. 4) are consistent with those based on synteny (figs. 1 and 2) and gene content (fig. 3). Previous studies have found that 16S rDNA does not provide good resolution for *Agrobacterium* and their related lineages, whereas *recA* is a more suitable marker for these bacteria (Costechareyre et al. 2010). Consistent with this finding, the 16S rDNA phylogeny was poorly resolved with low support (fig. 4*A*), whereas the trees based on *recA* (fig. 4*B*) and 1,467 single-copy genes conserved among these Rhizobiaceae strains (fig. 4*A*) both provided strong support that NCHU2750 is more closely related to *N. galegae* than to *A. tumefaciens*.

**Fig. 5.**
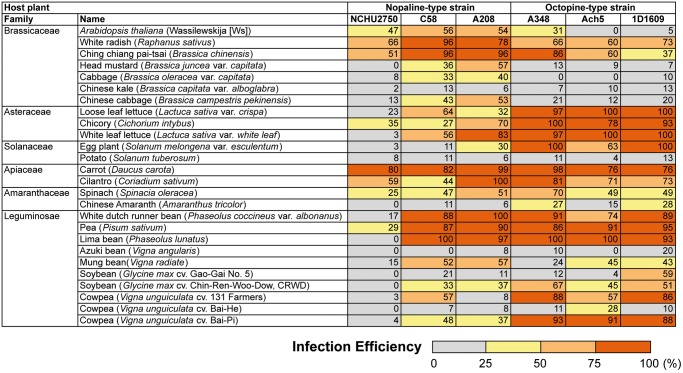
—Infection efficiencies against different host plants. Values are average percentages based on three independent experiments.

Our qualitative test confirmed that NCHU2750 could induce gall formation on rose (supplementary fig. S1, Supplementary Material online). The quantitative assays revealed that although NCHU2750 is capable of transient transformation in *Arabidopsis* and tumorigenesis in multiple plant hosts, its infection efficiency is lower than those representative *A. tumefaciens* strains (fig. 5). Comparison with C58 indicated that these two strains have nearly identical *virF* and *virE3*, which are two key genes that affect host range (Melchers et al. 1990; García-Rodríguez et al. 2006). This finding suggested that chromosomal background is also important in determining the phytopathogenicity, as has been demonstrated through reciprocal Ti plasmid exchange between C58 and 1D1609 (Palumbo et al. 1998).

In summary, the strain NCHU2750 is a novel lineage within Rhizobiaceae. Results based on chromosomal organization, gene content, and molecular phylogenies all support that this strain is closely related to *Neorhizobium*. Intriguingly, the three characterized *Neorhizobium* species all form root nodules with legume hosts (Lindström 1989; Wang et al. 1998; Lu et al. 2009; Mousavi et al. 2014), whereas NCHU2750 is a tumor-inducing phytopathogen. It is unclear whether this taxon should be proposed as a novel species within the genus *Neorhizobium* (e.g., *Neorhizobium tumefaciens*), or the representative of a novel genus. More detailed polyphasic investigation is necessary for establishing the taxonomy of this bacterium. The presence of an *Agrobacterium*-type Ti plasmid in NCHU2750 may be explained by horizontal acquisition, as one tumorigenic *A. tumefaciens* strain was isolated from the same gall (supplementary fig. S1, Supplementary Material online). Alternatively, this pTiNCHU2750 may be vertically inherited. More comprehensive taxon sampling in the *Allorhizobium*-*Agrobacterium*-*Neorhizobium* clade (Ormeño-Orrillo et al. 2015) is necessary for investigating this issue. Previous works have demonstrated that Ti plasmids may be artificially transferred to various Rhizobiales species and confer the ability to cause crown galls (Hooykaas et al. 1977; van Veen et al. 1988; Broothaerts 2005). The new strain characterized in this study further expanded phylogenetic distribution of naturally occurring Ti plasmids. Importantly, the capability of transforming plant hosts by this bacterium suggests that it may be used for future comparative studies with *Agrobacterium* to better understand the genetic determinants of host range among these bacteria.

## Supplementary Material

Supplementary data are available at *Genome Biology and Evolution* online.

## Supplementary Material

Supplementary Data
