# Supplementary material for: Presence of an Agrobacterium-Type Tumor-Inducing Plasmid in Neorhizobium sp. NCHU2750 and the Link to Phytopathogenicity
Source: Genome Biol Evol. 2018 Nov 6;10(12):3188–95. doi: 10.1093/gbe/evy249 (PMC6286910; doi:10.1093/gbe/evy249)
Supplement: Supplementary Data [file evy249_supp.zip › fig.gall.v2.pdf]

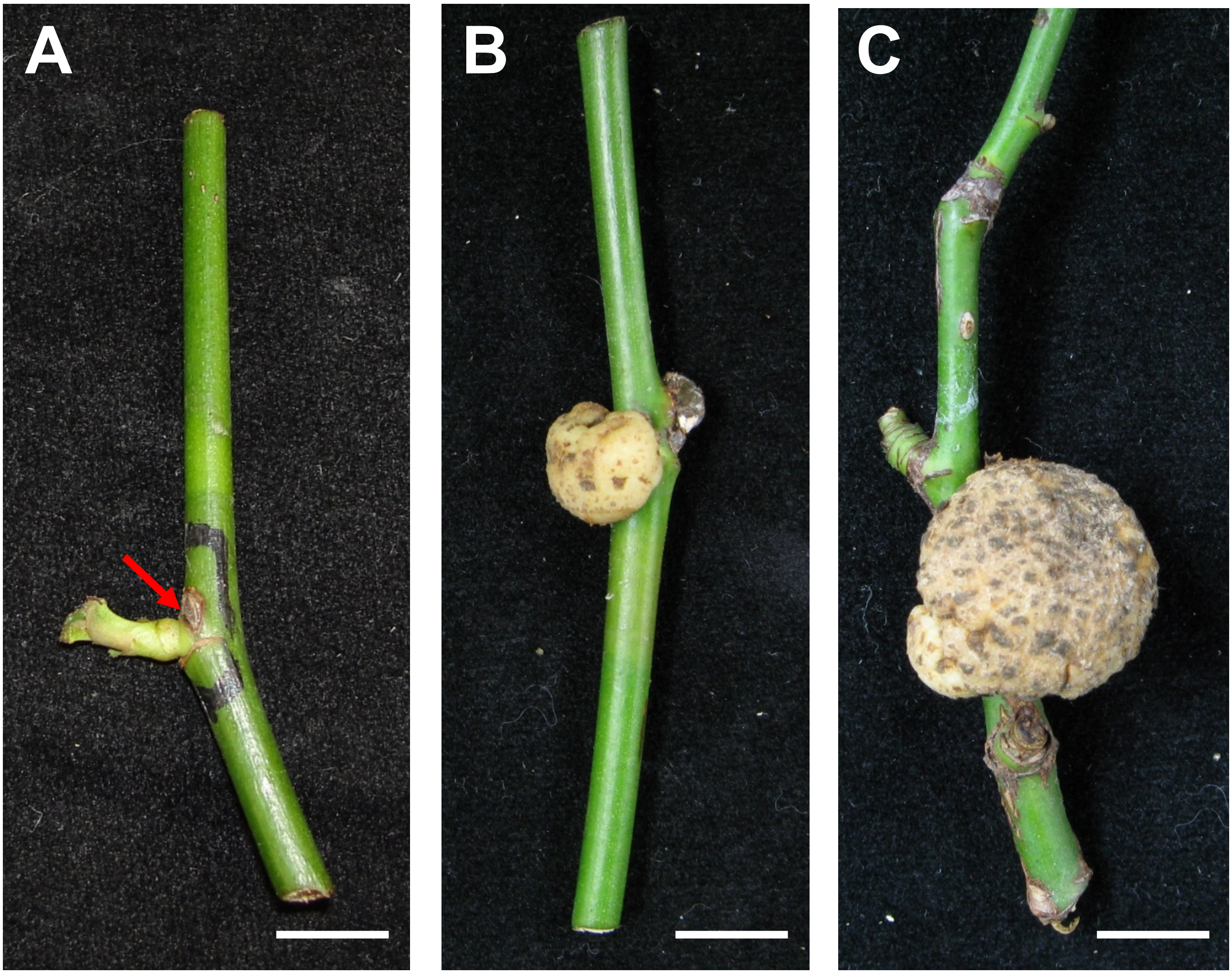

Gall formation on rose stem induced by artificial inoculation. (A) Negative control using sterile distilled water. Red arrowhead indicates the inoculation site. (B) Strain NCHU2750. (C) *Agrobacterium tumefaciens* strain A9, which was isolated from the same gall as NCHU2750. Photos were taken two months after inoculation. The pathogenic bacteria were isolated from the tumor tissues and confirmed by colony morphology and PCR to fulfill Koch's postulates. Scale bar: 1 cm.
