# Supplementary material for: Presence of an Agrobacterium-Type Tumor-Inducing Plasmid in Neorhizobium sp. NCHU2750 and the Link to Phytopathogenicity
Source: Genome Biol Evol. 2018 Nov 6;10(12):3188–95. doi: 10.1093/gbe/evy249 (PMC6286910; doi:10.1093/gbe/evy249)
Supplement: Supplementary Data [file evy249_supp.zip › Legends supplementary materials.docx]

**Supplementary Materials**

Table S1. Accession numbers of the sequences used in the molecular phylogenetic analysis.

Table S2. Homologous gene clusters among the four representative strains.

Figure S1. Gall formation on rose stem induced by artificial inoculation.
